# Maternal Serum Macrophage Inhibitory Cytokine-1 as a Biomarker for Ectopic Pregnancy in Women with a Pregnancy of Unknown Location

**DOI:** 10.1371/journal.pone.0066339

**Published:** 2013-06-18

**Authors:** Monika M. Skubisz, Jeremy K. Brown, Stephen Tong, Tu’uhevaha Kaitu’u-Lino, Andrew W. Horne

**Affiliations:** 1 Translational Obstetrics Group, University of Melbourne, Mercy Hospital for Women, Heidelberg, Victoria, Australia; 2 Monash Institute of Medical Research, Monash University, Clayton, Victoria, Australia; 3 MRC Centre for Reproductive Health, Queen’s Medical Research Institute, University of Edinburgh, Edinburgh, Scotland, United Kingdom; Institut Jacques Monod - UMR 7592 CNRS - Université Paris Diderot, France

## Abstract

**Background:**

Ectopic pregnancy (EP) occurs in 1–2% of pregnancies, but is over-represented as a leading cause of maternal death in early pregnancy. It remains a challenge to diagnose early and accurately. Women often present in early pregnancy with a ‘pregnancy of unknown location’ (PUL) and the diagnosis and exclusion of EP is difficult due to a lack of reliable biomarkers. A serum biomarker able to clearly distinguish between EP and other pregnancy outcomes would greatly assist clinicians in diagnosing and safely managing PULs. This study evaluates the ability of maternal serum macrophage inhibitory cytokine-1 (MIC-1) levels to differentiate between EP and other pregnancy outcomes in women with a PUL.

**Methods:**

Sera were collected from 120 women with a PUL at first clinical presentation and assayed for MIC-1 by ELISA. Results were classified according to ultimate pregnancy outcome and the discriminatory ability of MIC-1 to diagnose EP was assessed.

**Results:**

Serum MIC-1 levels were lower in women with histologically confirmed (definite) EP (dEP) (median 552 ng/mL; interquartile range (IQR) 414–693 ng/mL) compared to women with definite viable intra-uterine pregnancies (dVIUPs) (722 ng/mL; IQR 412–1122 ng/mL), and higher when compared to women with definite non-viable intra-uterine pregnancies (dNVIUPs) (465 ng/mL; IQR 341–675 ng/mL). MIC-1 levels were significantly higher in women with dEP compared to women whose PULs resolved without medical intervention (srPUL) (401 ng/mL; IQR 315–475 ng/mL) (p<0.003). There were no women with an ectopic pregnancy where serum MIC-1>1000 ng/mL.

**Conclusion:**

Serum MIC-1 levels in PUL were not able to categorically diagnose EP, however, MIC-1 could distinguish women with an EP that required medical intervention and those women whose PULs spontaneously resolved. A single serum MIC-1 measurement also excluded EP at levels above 1000 ng/mL. MIC-1 may play a role in the development of a combined assay of biomarkers for the diagnosis of EP.

## Introduction

Ectopic pregnancy (EP) occurs in 1–2% of pregnancies and remains a leading cause of maternal death in early pregnancy [1], [2]. The most common symptoms of EP are abdominal pain and/or vaginal bleeding, however, these are not specific to EP and can be consistent with a range of pregnancy outcomes, including on-going (definite) viable intrauterine pregnancies (dVIUPs) and miscarriages (definite non-viable intrauterine pregnancies – dNVIUPs). The challenge for the clinician is to be able to accurately distinguish between pregnancy outcomes in order to safely manage any woman presenting with these symptoms.

The diagnosis of EP relies on transvaginal ultrasonography (TVUS), and three quarters of EPs will be diagnosed at the initial scan (sensitivity of 73.9% and a specificity of 98.3%) [3]. When neither an intrauterine nor an EP can be visualized by TVUS, the woman is classified as having a pregnancy of unknown location (PUL) [4]. This requires further time and resource intensive follow-up, including serial serum human chorionic gonadotrophin (hCG) levels and repeat TVUS to determine pregnancy location and appropriate management [5], [6]. Between 7–20% of women with PUL are ultimately diagnosed with EP [7].

Recent research endeavour has focused on the potential for a serum biomarker to be able to accurately diagnose EP at the time of first presentation for women with PUL. A number of candidate molecules have been proposed, but as single markers, none are sufficiently discriminatory for diagnosing EP [8]. Some groups have attempted combining serum biomarkers in tests with improved diagnostic accuracy, ranging from 52.9–98% sensitivity and 92.4–100% specificity, however, these still require further validation before potentially being used in a diagnostic algorithm for EP [8].

Serum macrophage inhibitory cytokine-1 (MIC-1) is a divergent member of the TGF-ß superfamily of cytokines, originally shown to inhibit macrophages [9]. MIC-1 has been localized to the syncytiotrophoblast and shown to increase in serum across the first trimester of pregnancy [10], and is thought to increase the population of tolerogenic dendritic cells in the decidua [11]. We previously identified maternal serum concentrations of MIC-1 as a potential predictive biomarker of miscarriage [12]. We then validated this in a subsequent prospective cohort study of 782 women, which showed serum MIC-1 in early pregnancy to be significantly lower in women who went on to miscarry [multiples of the median (MOM) 0.63 (IQR 0.33–0.88) compared to women with successful pregnancy outcomes (MOM 1.00 (IQR 0.76–1.29) (p = <0.001), with an area under the curve (AUC) of 0.73 (95% CI 0.63–0.84) as a single biomarker predictor of miscarriage [13] ].

In this prospective cohort study, we assessed the ability of serum MIC-1 levels to diagnose EP amongst women with PULs who presented with abdominal pain and/or vaginal bleeding in early pregnancy.

## Results

We recruited 120 Caucasian women aged between 18–45 years and diagnosed with a PUL after presentation with abdominal pain and/or vaginal bleeding in early pregnancy. The outcomes of these pregnancies were classified according to the recent PUL consensus statement [4]. Table 1 details the definition and final breakdown of pregnancy outcomes for the cohort, with no differences demonstrated in their baseline characteristics (one-way ANOVA).

**Table 1:** Participant categorisation and baseline characteristics according to final pregnancy outcome (median ± SEM).

Group	Inclusion criteria	hCG (mU/mL)	PgP3 ELISA absorbance (450 nm)	Age (years)	Weight (kg)	BMI	n
dVIUP	Definite viable intrauterine pregnancy: TVUSS confirmation of intrauterine gestational sac with yolksac and embryo with cardiac activity.	4022±1904	0.53±0.18	32±1	74±3	27±1	17
dNVIUP	Definite nonviable intrauterine pregnancy: USS confirmation of intrauterine gestational sac with yolksac and/or embryo without cardiac activity seen prior to uterine evacuation.	6844±2017	0.34±0.15	28±1	70±4	26±2	8
dEP	Definite ectopic pregnancy: intervention prompted by adnexal mass on TVUSS or by abnormal risein serum hCG levels and confirmed at surgery and by histopathology.	1151±238	0.92±0.34	29±1	70±4	25±1	28
NP	Not pregnant: positive home pregnancy test result subsequently not confirmed by serum hCG measurement.	<5	0.72±0.42	26±2	70±8	27±3	26
srPUL	Spontaneously resolving PUL: PUL with spontaneous resolution of serum hCG levels.	428±114	0.70±0.17	32±1	74±4	28±1	27
tPUL	Treated persistent PUL: abnormal rise in serum hCG levels but no adnexal mass or IU sac seen onTVUSS after monitoring, managed medically with methotrexate.	400±188	0.73±0.73	32±4	83±15	28±5	3
pEP	Probable ectopic pregnancy: heterogenous adnexal mass or extra-uterine sac-like structure onTVUSS managed medically with methotrexate.	597±200	0.43±0.34	33±1	63±4	25±1	11

**Figure 1:**
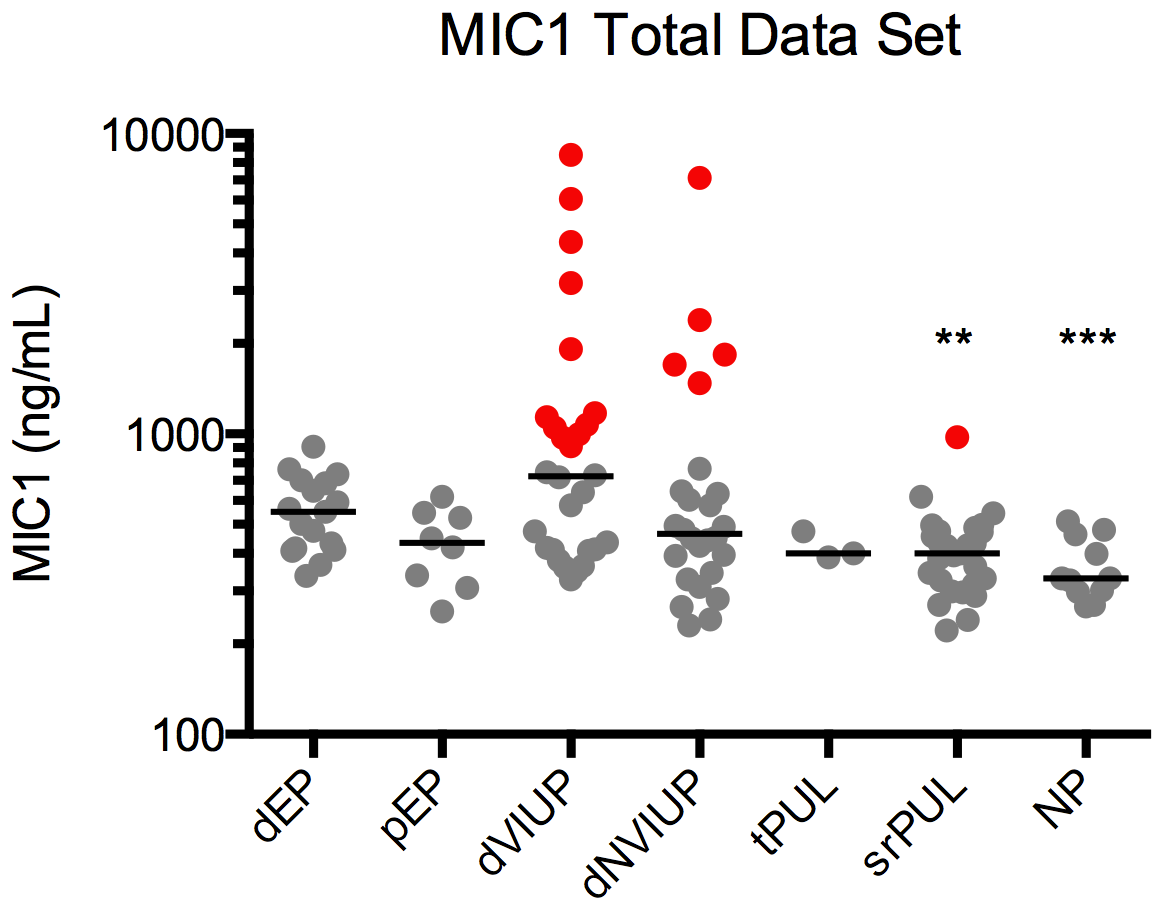
Serum MIC-1 levels in women at first presentation with a PUL, categorised according to final pregnancy outcome. Serum MIC-1 levels >1000 ng/mL exclude EP.

Serum MIC-1 levels were lower in women with histologically confirmed (definite) EP (dEP) (median 552 ng/mL; IQR 414–693 ng/mL) compared to women with definite viable intrauterine pregnancies (dVIUP) (722 ng/mL; IQR 412–1122 ng/mL), and higher compared to women with definite non-viable intrauterine pregnancies (dNVIUP) (465 ng/mL; IQR 341–675 ng/mL) and treated PULs (tPUL) (400 ng/mL; IQR 388–473 ng/mL). Levels were also higher in women with dEP than in women who were medically managed for probable EPs (pEP) (434 ng/mL; IQR 315–541 ng/mL). Additionally, MIC-1 levels were significantly higher in women with dEP compared to women with a PUL that resolved spontaneously (i.e. without medical intervention) (srPUL) (401 ng/mL; IQR 315–475 ng/mL) (p<0.003). Non-pregnant (NP) women were included as a control and their serum MIC-1 levels were significantly lower than in women with dEP (330 ng/mL; IQR 298–463 ng/mL) (p = 0.001) (Figure 1). Interestingly, a correlation was observed between serum MIC-1 and serum hCG levels in women with dVIUPs and dNVIUPs (Spearman; P<0.04 and P<0.03, respectively), but not in any other pregnancy outcome categories (P>0.35).

**Figure 2:**
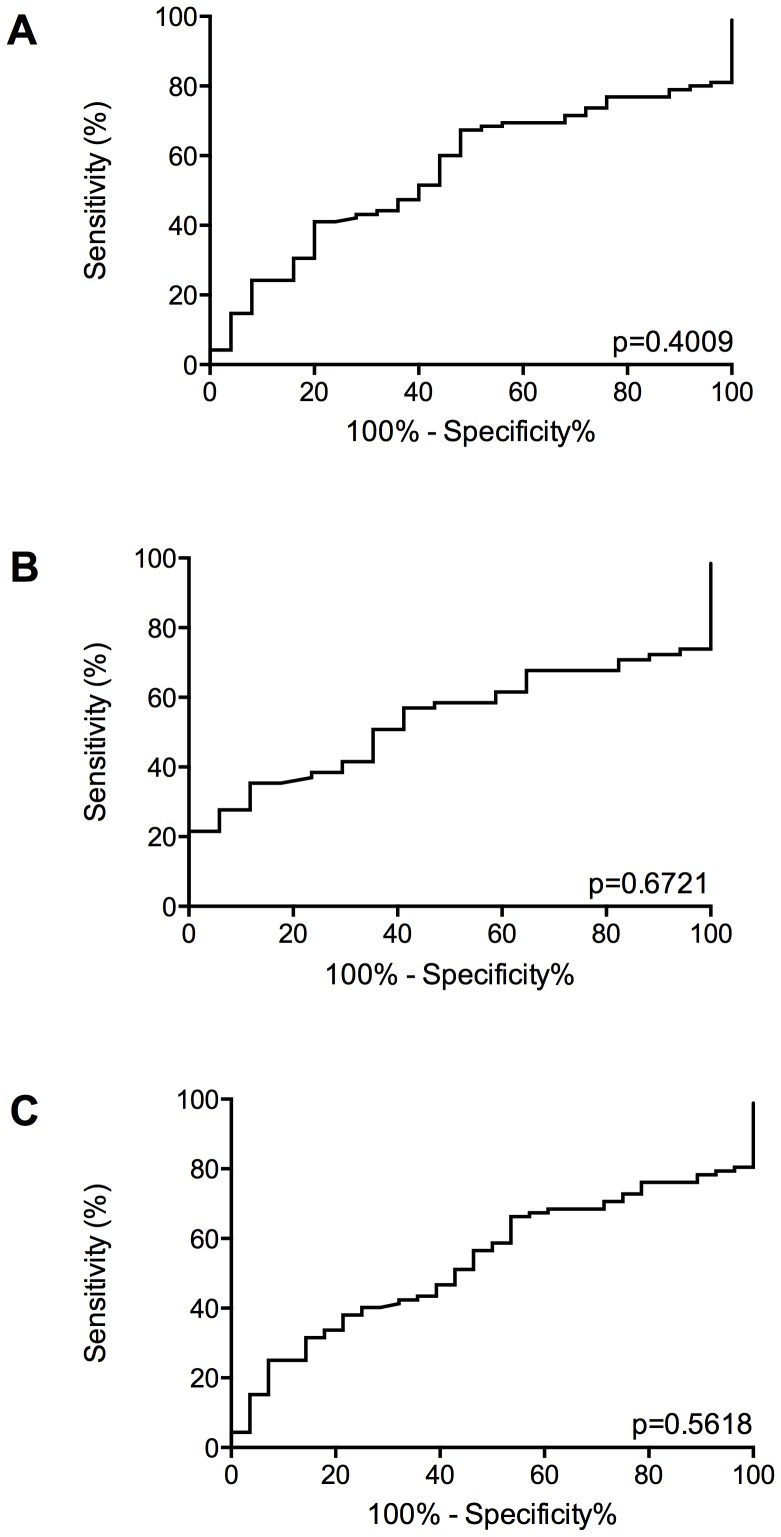
ROC Curve analyses of MIC-1 as a biomarker of EP. A) Comparing MIC-1 levels in women with EP (dEP and pEP) compared to all other pregnancy outcomes. B) Comparing MIC-1 levels in women with dEP compared to women with a definite non-EP outcome (dVIUP, dNVIUP, NP), excluding women with ambiguous pregnancy outcomes (srPUL, tPUL and pEP). C) Comparing MIC-1 levels in women requiring treatment (dEP, pEP and tPUL) compared to women who did not (dVIUP, dNVIUP, srPUL and NP).

ROC curve analysis suggests MIC-1 would have limited use in the diagnosis of EP as a single biomarker (AUC = 0.5547; p>0.4) (Figure 2a). Removal of ambiguous PUL outcomes (srPUL, tPUL and pEP) did not improve the performance of MIC-1 as a biomarker of EP (AUC = 0.5335; p>0.6) (Figure 2b), nor did grouping pregnancy outcomes by those that required treatment (dEP, pEP and tPUL) compared to those that did not (dNVIUP/dVIUP/NP/srPUL) (AUC = 0.5363; p>0.5) (Figure 2c). A single serum MIC-1 measurement, however, excluded EP at levels above 1000 ng/mL (Figure 1) (positive predictive value (PPV) 100%, negative predictive value (NPV) 24%, sensitivity 17% and specificity 100%; *P*<0.05).

## Discussion

In this study, we demonstrated that serum MIC-1 measurement has some potential as a diagnostic biomarker of EP in women with a PUL. We prospectively recruited 120 women with an interim diagnosis of PUL, who presented with symptoms of abdominal pain and/or vaginal bleeding after a positive home pregnancy test. Importantly, serum samples were collected at the time of first presentation and not at the time of diagnosis, as would reflect true clinical practice. We followed these women until complete resolution of their pregnancies and classified their outcomes according to the PUL consensus statement [4]. Whilst the number of women in each pregnancy outcome category was relatively small, the full range of PUL outcomes were represented in the cohort. We additionally verified that previous exposure to C. *trachomatis* did not influence MIC-1 levels in the sera of participants. Hence this study robustly tests the potential of serum MIC-1 levels as a biomarker of EP.

Although this study showed that a single serum MIC-1 level is not sufficiently discriminatory to *diagnose* EP, serum MIC-1 level measurement may help to *exclude* EP. No woman participating in this study had an EP where her serum MIC-1 level >1000 ng/mL. Hence, clinicians could use a serum MIC-1 cut-off, above which a woman is very unlikely to have an EP, to help determine which women with PULs can be safely followed up as outpatients. Furthermore, serum MIC-1 levels were significantly higher in women with dEPs compared to women with srPULs. Therefore clinicians could potentially use serum MIC-1 levels in PUL to help determine which women are likely to require treatment. Both these approaches, however, would require further validation before being used in a clinical management algorithm for PUL.

The inability of any single biomarker being able to sensitively and specifically diagnose EP has led some groups to combine serum biomarkers in an attempt to improve diagnostic accuracy. In particular, Rausch notes that biomarkers with varied biological actions seem to perform best together [8]. This reflects an attempt to identify numerous deviations in physiological processes common to both intrauterine and ectopic pregnancy, the sum of which may allow a combined biomarker panel to predict location. To date, the best combination of biomarkers (progesterone, vascular endothelial growth factor (VEGF), inhibin A and activin A) has achieved a sensitivity of 98% and a specificity of 100%, but only in 42% of the cohort that was able to be characterised [14]. This panel of biomarkers still requires external validation in a prospective cohort to determine its clinical viability.

MIC-1, Placental Growth Factor (PlGF) and fms-related tyrosine kinase 1 (flt1) levels are all lower in EP compared to dVIUP and higher compared to dNVIUP, [15], [16] however, their reported test performance as single biomarkers for the diagnosis of EP are difficult to compare due to different cut-offs and outcome groupings used in their assessment [8]. What is apparent though, is that MIC-1, PlGF and flt1 all correlate with pregnancy viability. VEGF in comparison, is elevated in EP compared to dVIUP [14], [17], and in terms of biomarker performance, can distinguish between EP and dVIUP and EP and dNVIUP with 100% specificity at cut-offs of 200 pg/mL and 174.5 pg/mL, respectively [18], [19]. As MIC-1>1000 ng/mL excludes EP and VEGF >200 pg/mL diagnoses EP, both with 100% specificity, the use of these two biomarkers in a diagnostic algorithm may prove clinically useful, provided their physiological relationship is not simply diametrically opposed. This would require further investigation.

Previous studies in early pregnancy show a correlation between low maternal serum MIC-1 levels and non-viable pregnancies [12], [13]. In this study, serum MIC-1 levels were lower in women with dEP compared to women with dVIUP, consistent with the likely insufficient trophoblastic invasion of an EP and its limited viability within the Fallopian tube environment. In contrast, serum MIC-1 levels were significantly higher in women with dEP compared to women with a srPUL, suggesting that serum MIC-1 levels can discriminate between a pregnancy that continues to grow and requires treatment compared to one that is demising.

Furthermore, a correlation was noted between serum MIC-1 and serum hCG levels in women with dVIUPs and dNVIUPS, but not in any other pregnancy outcome categories. MIC-1 and hCG were both lower in women with dNVIUPs compared to women with dVIUPs (P = 0.1 and P<0.02, respectively). This suggests MIC-1 may be up-regulated in viable pregnancy, to help mediate an appropriate immune response within the uterine environment. Hence serum MIC-1 levels in this study continue to correlate with relative pregnancy viability.

In conclusion, as a single biomarker, a serum MIC-1 level at the time of TVUS diagnosis of a PUL is not sufficiently discriminatory to diagnose EP. Serum MIC-1 levels do, however, correlate with pregnancy viability and may be used as a cut-off for exclusion of EP and for determination of which women with a PUL will require treatment. Serum MIC-1 levels may therefore be useful as part of a management algorithm for PUL and could likely form part of a multiplex biomarker assay, to assist clinicians in diagnosing EP.

## Materials and Methods

### Patient Samples

This study was approved by the Lothian Research Ethics Committee (LREC 04/S1103/20 and 09/S1103/39). Written, informed consent was obtained from all participants. Women presenting with abdominal pain and/or vaginal bleeding after a positive home pregnancy test and in whom a TVUS was not able to locate the pregnancy (i.e. PUL) were recruited. Whole blood was collected from participants and after clotting for 2 hrs at room temperature, the sera were collected and stored and −80°C in multiple aliquots. Participants were followed up and their ultimate pregnancy outcomes classified according to the recent PUL consensus statement [4].

### Ultrasound Assessments

TVUSs were performed using the Toshiba Aplio XG machine by a team of trained, qualified and experienced sonographers.

### MIC-1 ELISA

Sera were assayed using the GDF-15 (MIC-1) Duoset ELISA kit (R&D systems, Abingdon, UK) according to the manufacturer’s instructions. Further details and performance parameters are available from the manufacturer (www.rndsystems.com/pdf/DY957). The ELISA was performed in one run, with samples scrambled throughout the plate. The scientist performing the ELISA was blinded to clinical groupings of samples.

Because current or past infection with Chlamydia *trachomatis* is strongly correlated with risk of EP, and MIC-1 is a cytokine whose expression may be influenced by other inflammatory and/or infective processes in the body, we also measured participant’s serum PgP3 antibody titres as a marker of previous C. *trachomatis* exposure by ELISA, as previously described [20].

### Statistical Analysis

Statistical analysis and ROC curves were generated using Prism 5.0 (GraphPad Software, La Jolla, USA).
